# Herbivore-specific plant defense responses to a pre-attack risk cue

**DOI:** 10.1371/journal.pone.0358257

**Published:** 2026-10-07

**Authors:** Eirette M. Santiago, Katherine M. Overstrum, Brooke A. Pellegrini, Abigail F. Gill, Reagan M. McGee, Faye Santaniello, Jack S. Whitney, Colin M. Orians, John L. Orrock, Evan L. Preisser

**Affiliations:** 1 Department of Biological Sciences, University of Rhode Island, Kingston, Rhode Island, United States of America; 2 Department of Biology, Tufts University, Medford, Massachusetts, United States of America; 3 Department of Integrative Biology, University of Wisconsin, Madison, Wisconsin, United States of America; Umeå Plant Science Centre, Umeå University, SWEDEN

## Abstract

Cues of potential herbivory, such as the locomotion mucus from gastropod herbivores, may influence germination and growth. Because it remains unclear whether use of gastropod cues to initiate defense is a widespread phenomenon in plants, we examined how mucus from multiple gastropod herbivores (*Arion subfuscus, Novisuccinea ovalis, Ambigolimax valentianus, Cornu aspersum, Deroceras laeve,* and *Deroceras reticulatum*) affects *Brassica nigra* germination and seedling biomass. In a two-year replicated experiment, germination timing exhibited species-specific responses and varied across years. Exposure to *Ar. subfuscus* mucus consistently accelerated germination timing relative to the water-only control, while *Am. valentianus* and *C. aspersum* mucus increased germination speed in one but not both years. Mucus from *D. laeve*, *D. reticulatum* and *N. ovalis* did not affect germination speed. Mucus exposure did not affect the proportion of seeds germinating in either year. Seedling biomass responses to mucus exposure were limited and varied between years: only *C. aspersum* affected biomass in both years, reducing biomass in the first year and increasing it in the second relative to the control. Exposure to *D. laeve* mucus reduced seedling biomass by 29% in the first year, whereas mucus from its congener *D. reticulatum*, tested in the second year, had no effect. Mucus from the remaining species did not affect seedling growth. Together, these results indicate that exposure to some gastropod mucus treatments can alter *B. nigra* germination timing and early seedling growth, with responses varying among gastropod species and between years. The lack of consistent responses across several gastropod species suggests that plants may perceive or respond to herbivore cues unevenly, potentially reflecting differences in mucus chemistry among species or environmental conditions influencing plant sensitivity to these cues. Our findings highlight the complexity of gastropod-plant interactions and suggest that environmental or physiological conditions play an important role in shaping plant responses to herbivore-associated chemical cues.

## Introduction

Plants exhibit a variety of defenses against herbivores [1,2]. These include constitutive defenses, which are always present, and inducible defenses activated in response to an attack [3,4]. Because producing anti-herbivore compounds can reduce resource allocation to growth, natural selection is expected to favor inducible defenses that allow plants to limit defensive investment until herbivores are present [5–7]. Balancing this growth-defense tradeoff selects for plants that can effectively evaluate threats before mounting a costly defensive response [8,9]. The modular nature of plants means that low-to-moderate levels of herbivory pose relatively little risk to the growth and fitness of mature individuals [10]. Most plants therefore minimize the cost of defense by activating defensive traits only following herbivore attack [9]. Seeds and young seedlings, however, have relatively little capacity for compensatory growth following herbivory [6]. As a result, natural selection is expected to favor plants that can detect and respond to pre-attack cues of herbivory during these vulnerable germination and seedling stages.

Kairomones are chemical signals produced by one species which are detected by and provide a benefit to an unintended ‘receiver’ species [11,12]. These include predator-produced hormones, pheromones, and metabolic byproducts that can be detected by and benefit their prey [13]. Snail locomotion mucus, for instance, leads to increased defense in *Solanum lycopersicum,* and herbivore frass can play a role in defense induction for a variety of agricultural plants [14–18]. Pellegrini et al. (2024) found that *Brassica nigra* seeds exposed to mucus from *Arion subfuscus* germinated faster than control seeds, producing smaller seedlings that were less palatable to herbivores. This result was consistent with work showing that *B. nigra* seeds and seedlings exposed to snail mucus had lower biomass but experienced less herbivore damage than non-exposed control plants [19]. Because terrestrial gastropod herbivores occur worldwide, exploring whether plants respond similarly to other gastropod species may provide important insights into large-scale variation in plant defense and growth. Currently, however, the impact of gastropod kairomones on seeds and seedlings has only been assessed for a handful of plant-herbivore interactions. Evidence that plants can perceive gastropod mucus also comes from molecular studies. Mucus from several slug and snail species has been shown to induce salicylic-acid associated defense gene expression in *Arabidopsis thaliana* [20]. Together these studies suggest that gastropod mucus may contain substances that act as kairomones for at least some plant species.

An array of herbivorous gastropods is found at high densities in mesic and hydric fields and forests throughout the northeastern United States [21]; exposure to their kairomones may impact plant performance traits. We assessed the responses of *B. nigra* seeds and seedlings to the mucus of six gastropod species that co-occur in the southern New England portion of this region. Our aim was to test whether mucus from multiple gastropod species alters germination timing and seedling growth in a co-occurring plant species. Based on previous work, we hypothesized that exposure to the mucus of each gastropod would increase germination speed but reduce seedling biomass*.*

## Methods

We replicated the same experiment in two consecutive years (early fall 2024 and 2025). Because every attempt was made to use identical methods between the two years, we describe the methodology for both below and identify differences between the 2024 and 2025 protocols wherever necessary.

### Gastropod species

Mucus from six gastropod species was used in this experiment: four slugs, (*Arion subfuscus*, *Deroceras laeve, Deroceras reticulatum,* and *Ambigolimax valentianus)* and two snails (*Cornu aspersum* and an amber snail provisionally identified as *Novisuccinea ovalis*). The slug *Arion subfuscus* is native to Europe but has been in North America for over a century and is widely distributed in the eastern United States [22–25]. It is currently the most abundant slug in New England fields and forests [26,27]. *Deroceras reticulatum*, another species introduced from Europe, is the dominant slug pest in local gardens and agricultural systems [28–32]. *Deroceras laeve* is one of the few widespread slug species considered native to parts of North America. It has worldwide distribution and is found in a variety of natural and anthropogenic habitats [29]. *Ambigolimax valentianus*, a common greenhouse pest, is native to the Iberian Peninsula and was first recorded in North America in 1944 [21,33]. The brown garden snail *Cornu aspersum* (formerly *Helix aspersa)* is native to Europe but is now distributed worldwide; it was introduced to North America over a century ago [34–36] and is found throughout the northeastern states [37]. The oval amber snail *Novisuccinea ovalis* is one of at least 20 species of amber snails (gastropod family Succineidae) native to the eastern United States. While this species is common in New England [38] and we are confident with this determination, it is important to note that snails in this family are difficult to identify beyond the family level without genetic analysis [39–44]. Because we could not conduct genetic analyses, their identification as *N. ovalis* should be considered provisional.

We collected *Ar. subfuscus, D. reticulatum*, and *N. ovalis* from a meadow at the University of Rhode Island’s East Farm, and *Am. valentianus* and *Deroceras laeve* from the greenhouse grounds (Kingston RI) in early fall 2024 and early fall 2025; the *C. aspersum* used in the experiment came from a colony collected in 2023 and subsequently maintained in the lab. The gastropods were segregated by species and held in 38 L glass terrariums; each terrarium contained a plastic drainage tray at the bottom topped with 10 cm of soil and then a layer of forest-collected dried leaves and bark. They were maintained on a diet of organic lettuce (*Lactuca sativa*), carrots (*Daucus carota*), and cabbage (*Brassica oleracea*), with Sepia-King cuttle bones (Sepia King LLC, Vietnam) provided for calcium supplementation. Food was replaced three times per week, and each terrarium was misted when necessary.

No permits were required for field site access or specimen collection. The collection site is owned by the University of Rhode Island and is available for use by University of Rhode Island students for research activities. The study involved the collection of terrestrial gastropod invertebrates (slugs and snails) in Rhode Island, and no state or federal collection permits were required for the species included in the study.

### Plant species

We chose the black mustard *Brassica nigra* for two reasons. First, prior work has shown that mucus alters its performance and resistance (Pellegrini et al. 2024). Second, while native of Eurasia [45–47] it has spread worldwide and is found throughout New England [48]. Importantly, it is an annual forb that thrives in riparian, field/grassland, and anthropogenically modified systems [45,49] where gastropods are abundant. *Brassica nigra* seeds were obtained from Outsidepride Seed Source LLC (Independence, OR).

### Mucus collection

The night before mucus collection, mature individuals of each species were removed from their terraria and placed in a 5.7 L plastic container with only water provided (one species per container). Petri plates for mucus collection were prepared the next morning by adding 1 g of dried, sifted potting soil (Miracle-Gro) to 60-mm plates, mixing it with 4 g of water, and spreading the mixture evenly across the bottom of each plate. After the plates were prepared, all gastropods were quickly rinsed with water and placed on moistened paper towels for 10 minutes to remove any remaining debris. Varying numbers of individuals from each species (see below) were then added to each single-species plate; each plate was topped with aluminum foil to encourage species to move around on the soil rather than sheltering under the lid. This was true for all species but *C. aspersum*, whose large size required the plates to be covered with 60-mL Solo condiment cups instead. On the basis of data from pilot experiments, in 2024 we varied the number of individuals per plate per species to ensure a minimum of 50% mucus coverage on the soil as follows: *Ar. subfuscus* (2 slugs, ~ 2.32 g total weight), *D. laeve* (3 slugs ~ 1.18 g), *Am. valentianus* (2 slugs ~1.01 g), *C. aspersum (*1 snail ~ 5.72 g), *N. ovalis* (4 snails, ~ 0.86 g). The corresponding values for the 2025 experiment were as follows: *Ar. subfuscus* (2 slugs, ~ 2.18 g total weight), *D. reticulatum* (2 slugs ~ 1.11 g), *Am. valentianus* (2 slugs ~1.36 g), *C. aspersum (*1 snail ~ 3.70 g), *N. ovalis* (4 snails, ~ 0.76 g). Both the 2024 and 2025 experiments included a control treatment with plates that contained moistened soil but no gastropod mucus. In 2024 each treatment (= species) was replicated in 16 plates for *Am. valentianus* and 17 plates for remaining treatments. In 2025 there were 17 control replicates, 18 replicates for *D. reticulatum* and *N. ovalis*, and 20 replicates for *Ar. subfuscus*, *Am. valentianus*, and *C. aspersum*.

Once all individual gastropods had been placed in their plates, the plates from different species were unsystematically interspersed and held in a dark cabinet for 24 hours at 23 °C. Once all gastropods had been removed and returned to their respective terraria, the plates were capped tightly, placed in freezer bags, and frozen at −20°C until use. Previous research has shown that frozen gastropod mucus maintains its kairomone activity for at least 100 days [50]. We also conducted a series of pilot experiments in summer 2024 confirming that fresh and previously frozen mucus from the tested gastropod species had the same effect on *B. nigra* seeds.

### Germination experiment setup

Five days after freezing, the plates were removed from the freezer and allowed to come to room temperature. A standard Zenpore qualitative grade 4 filter paper disk was placed on top of the soil in each plate and moistened with 2 mL of distilled water. Thirty *B. nigra* seeds were then placed on top of each filter paper; this prevented direct contact while exposing them to chemical cues diffusing from the soil. After replacing the lid on each plate, we interspersed plates from different treatments in an unsystematic manner and divided the plates between two 15 L plastic containers. Both containers were placed in a growth chamber in darkness at 21 °C.

### Seed germination and planting

Seed germination was assessed using a Petri plate germination assay. Starting 12 hours after the plates were placed in the growth chamber, and continuing every two hours thereafter, each plate was checked for germinated seeds (defined as radicle emergence). The seed checks ended at hour 30 in 2024, since most germination had ended by that point. In 2025, germination was slower than in 2024, and monitoring continued until all treatments had a mean of at least 20 germinated seeds per plate. Because the observation period was designed to capture the main phase of germination rather than to confirm absolute final germination after an extended plateau, we interpret treatment effects primarily as differences in germination timing rather than definitive differences in final germination success. The first two seeds to germinate from each plate were planted individually at a depth of 2 cm in 14-cm-deep pots (“Cone-tainers”; Stuewe & Sons, Inc., Tangent, OR, USA) filled with organic Pro-MIX garden soil (Quakertown, PA, USA) to ensure that at least one seedling was available from each independent Petri-dish replicate for the seedling growth assay. The planted seeds were held in a growth chamber under a 12:12 L:D regime at 23 °C (light) and 20 °C (dark). Plants were watered with distilled water every day for the first week and every other day afterwards. The tubes were placed in 7 x 7 trays so that each treatment occurred once in each row and once in each column. A seventh “mock” set of tubes was included to complete the grid. Tray positions were rotated daily, and tube positions were shifted within trays to minimize positional effects.

Seedling growth was assessed at harvest. In 2024 plants were harvested 10 days after planting. In 2025, we grew the plants longer. Specifically, seedlings were first held in a growth chamber as described above for five days, then moved to a greenhouse with natural light and 70% light supplementation from grow lights on a 12:12 L:D regime until being harvested after 20 days.

Each plant was harvested by cutting its shoot at the soil line and placing it in an individual 6 x 9 cm coin envelope. In 2024, each root system was excavated separately, washed carefully to remove soil particles, and left to air dry before being placed in a coin envelope. Because analysis of the 2024 data revealed no effect of mucus cue on root biomass (see below), in 2025 we harvested, dried, and weighed only the shoot portion of each plant. The envelopes were dried for 48 hours at 60 °C before weighing.

### Statistical analysis

To explore the effect of mucus on the speed and magnitude of seed germination, we initially examined the germination data using failure-time analyses (mixed-effects Cox proportional hazards models), modeling each year separately because of differences in the duration of the experiment in 2024 and 2025. Because model diagnostics indicated that the proportional hazards assumption was not supported for either mixed-effects Cox model (hazard lines crossed among treatments as the experiments proceeded), we explored additional analytical options, including mixed-effect accelerated failure-time models, generalized linear mixed models (GLMM) with a binomial response distribution, and restricted mean survival time models that incorporated random effects [51,52]. We opted to use binomial GLMM as our primary analytical approach because it allowed us to explicitly incorporate interactions between time and treatment. Importantly, our conclusions are robust to the analytical approach used, as we obtain qualitatively similar results from mixed-effect accelerated failure time analyses and restricted mean survival time models (although the latter examines a particular point in time). The model specified mucus treatment as a fixed effect, the hour since the experiment was initiated as a covariate and included a cubic term for hour. We modeled time as non-linear because preliminary analyses indicated a non-linear relationship between probability of germination and the hour since the experiment was initiated. The cubic relationship was a significantly better fit than linear or quadratic based on comparison of AICc and log-likelihood tests (all p-values <0.001). Germination at each observation time was analyzed as the number of germinated seeds per dish; we modeled each replicate plate as a random effect, using an autoregressive covariance structure to accommodate repeated observations collected on the same plate. All GLMM analyses were conducted using the glmmTMB package [53] in R [54]. We use the DHARMa package to assess whether under- or overdispersion existed in fitted GLMM models. Following any significant main effects we used Tukey’s test to adjust for multiple comparisons when using pairwise comparisons to evaluate significant main effects [55]. We examined the effect of mucus on seedling growth (measured as seedling biomass) using a linear mixed model that specified treatment as a fixed effect and box as a random effect. Following a significant main effect of treatment, Dunnett’s test was used to compare each mucus treatment with the control at α = 0.05. These analyses were conducted using JMP (18.1.1) [56].

## Results

### Germination timing and cumulative germination

In 2024 (Figs 1A–1E and 2A), there was a significant main effect of seed treatment (χ² = 49.51, 5 d.f., p < 0.0001), a significant cubic effect of time (χ² = 3953.3, 1 d.f., p < 0.001), and a significant interaction between seed treatment and time (χ² = 38, 5 d.f., p < 0.001). Across the observation period, germination differed among treatments relative to the water control. Germination speed was significantly higher for seeds exposed to *Ar. subfuscus* (linear contrast, z = 6.01, p < 0.001), *Am. valentianus* (z = 4.83, p < 0.001), and *C. aspersum* (z = 2.97, p = 0.036). At hour 20, cumulative germination was 91% higher in the *Ar. subfuscus* treatment, 71% higher in the *Am. valentianus* treatment, and 41% higher in the *C. aspersum* treatment than in the control. There was no difference in the probability of germination between control seeds and those exposed to *D. laeve* (z = 1.51, p = 0.660) or *N. ovalis* (z = 2.23, p = 0.223). The significant interaction between time and seed treatment reflects the time-sensitive nature of seed-treatment effects: treatment with *Am. valentianus* and *C. aspersum* led to significant increases in germination probability that were apparent early (i.e., 16 hours after the start of the experiment) as well as at the midpoint of the experiment (20 hours), whereas the *Ar*. *subfuscus* treatment only increased germination probability near the midpoint of the experiment. None of the treatments affected germination probability near the end of the experiment (26 hours) when most germination had occurred, indicating that the treatments differed in germination timing rather than final germination percentage. Simulation-based tests of dispersion indicated that there was no significant indication of under- or overdispersion (dispersion statistic = 0.99; p = 0.56).

**Fig 1:**
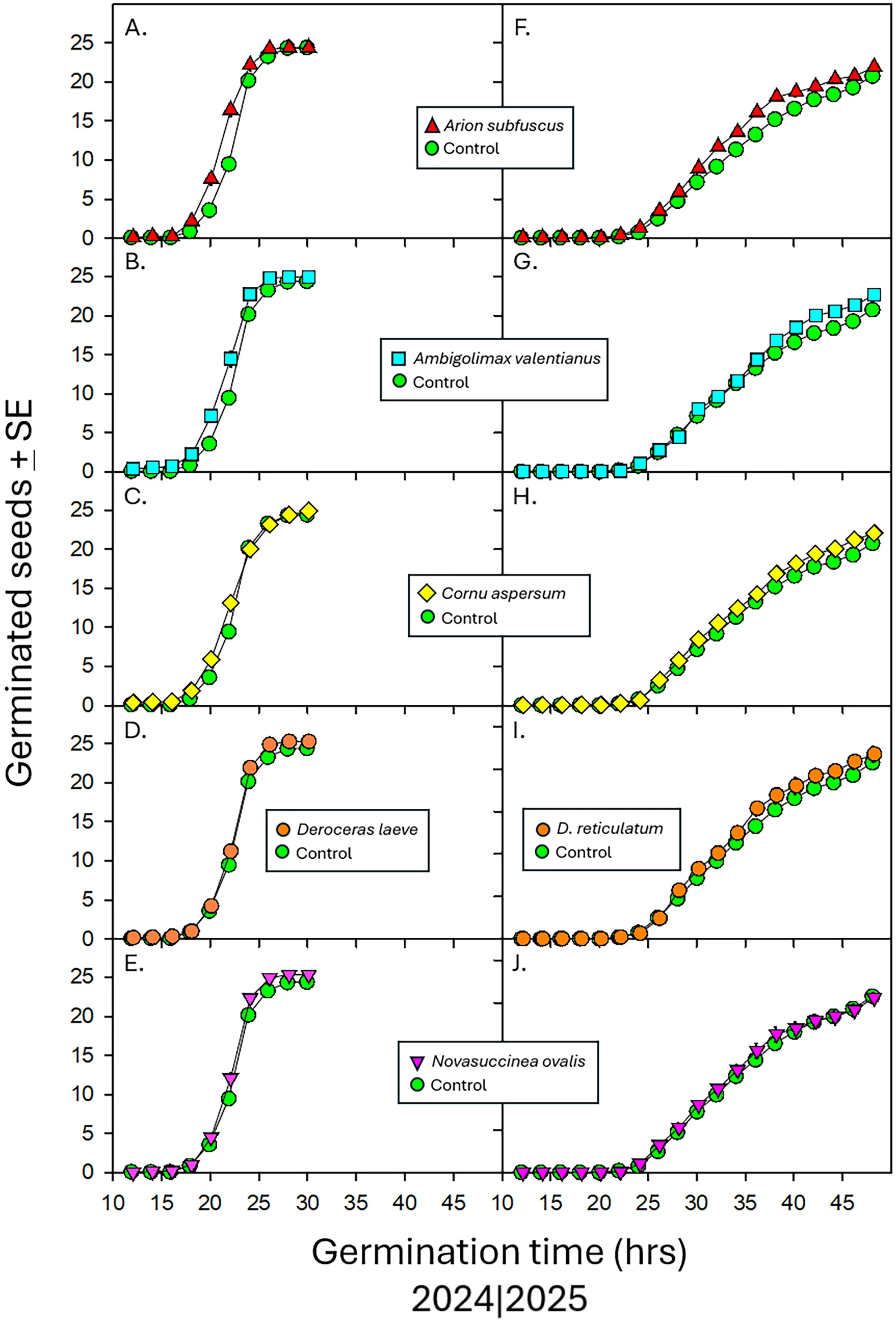
Germination of *B. nigra* seeds exposed to gastropod locomotion mucus in 2024 (left column) and 2025 (right column). Each panel presents mean cumulative germination (± SE) over time across multiple replicate Petri dishes for seeds exposed to locomotion mucus of each gastropod species compared to non-mucus control seeds (green circles).

**Fig 2:**
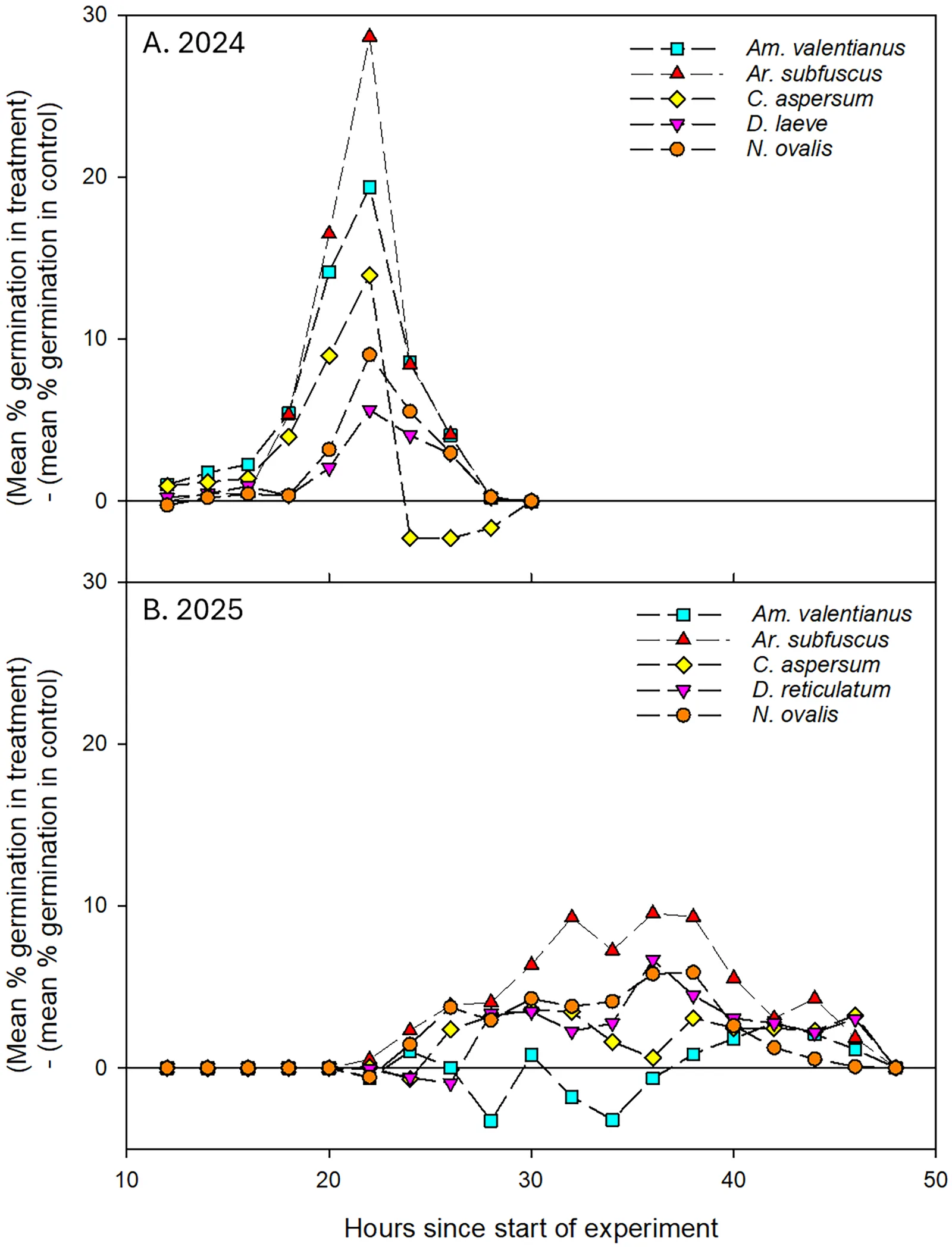
Difference in *B. nigra* germination percentage between the mean values of each treatment (locomotion mucus from different gastropod species) and the water-only control across the duration of the experiment. Positive values indicate that mean cumulative germination percentage was higher in that treatment than the control for that sampling point. Blue squares: *Am. valentianus*, red triangles: *Ar. subfuscus*, yellow diamonds: *C. aspersum*, inverted fuchsia triangles: *D. laeve* (2024) or *D. reticulatum* (2025), orange circles: *N. ovalis*.

In 2025 (Fig 1F–1J and 2) there was a marginally significant main effect of seed treatment (χ² = 9.87, 5 d.f., p = 0.08), a significant cubic effect of time (χ² = 3600.6, 1 d.f., p < 0.001), and no interaction between seed treatment and time (χ² = 3.84, 5 d.f., p = 0.57). There was a trend towards a higher proportion of seeds germinating in the *Ar. subfuscus* treatment compared to the water control *(*linear contrast, z = 2.72, p = 0.07); none of the other treatments differed from the control in their effect on cumulative germination probability (all p > 0.68). Although not significant at α = 0.05, there the dispersion statistic suggested slight underdispersion (dispersion statistic = 0.96, p = 0.064) for the 2025 model.

### Seedling biomass

Aboveground seedling biomass differed among treatments in 2024 (Fig 3A: F with 5 and 180.7 d.f. = 20.6, p < 0.001) and 2025 (Fig 3B: F with 5 and 210.2 d.f. = 2.47, p = 0.033). In 2024, seeds exposed to *D. laeve* and *C. aspersum* mucus grew into seedlings that were 29% and 71% smaller, respectively, than control seedlings (Dunnett’s test, p < 0.05); seedlings from the other three treatments did not differ from control plants. While belowground biomass differed between treatments (F with 5 and 179.2 d.f. = 3.67, p = 0.004) in 2024, none differed significantly from the control. In 2025, *C. aspersum* mucus-exposed seeds grew into seedlings with 26% more aboveground biomass than control seedlings (Dunnett’s test, p < 0.05); other treatments did not differ from the control.

**Fig 3:**
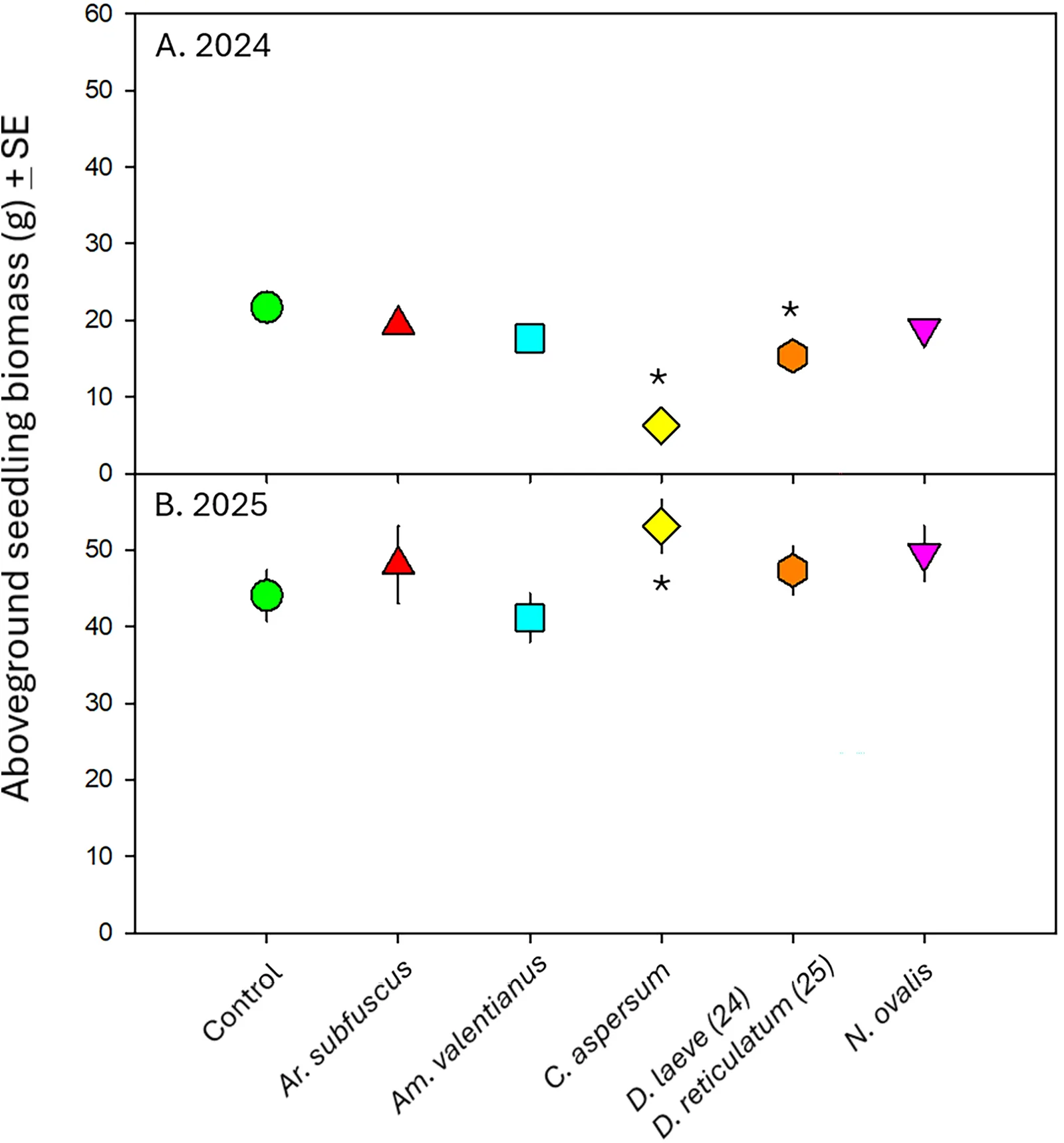
Effects of different treatments on aboveground plant biomass in 2024 (A) and 2025 (B). Asterisks indicate a statistical difference (p < 0.05) between the control seedlings (green circle) and those exposed to locomotion mucus of different gastropod species (Dunnett’s test).

## Discussion

Defense against herbivores may be particularly important when plants are young because the probability of surviving an attack is likely low for a seedling [6,10]. Herbivore-associated cues, such as those present in gastropod locomotion mucus, can signal future herbivory risk and some seeds and seedlings have been shown to respond to mucus exposure at the expense of growth [19,52]. Across two consecutive years, we conducted similarly designed experiments to test whether exposure to mucus from six herbivorous gastropod species influenced the germination and early seedling development of the co-occurring introduced plant, black mustard *B. nigra*. Our results indicate that plant responses to gastropod mucus are highly variable, differing among herbivore species and between years, with clear evidence for effects on germination timing but not on final germination percentage.

Only one gastropod species, *Ar. subfuscus*, increased germination speed in both years, although the effect in 2025 was only marginally significant. Both *Am. valentianus* and *C. aspersum* mucus increased germination speed in 2024 but had no detectable effect in 2025. Mucus from *N. ovalis* showed no detectable effect on germination speed in either year. Mucus from *D. reticulatum* and *D. laeve* also failed to produce a response. These findings suggest that responses of *B. nigra* to gastropod-derived cues vary strongly between years, a pattern aligned with other work showing that ecological interactions are shaped by environmental variability [57,58]. Seedling biomass showed little evidence of repeatable responses to mucus exposure. Although *C. aspersum* mucus affected biomass in both years, the fact that it alternately reduced (2024) and increased (2025) biomass suggests that the response was not reproducible. The differing patterns between germination speed and seedling biomass suggest that any responses to gastropod mucus are likely to be strongly influenced by environmental or physiological conditions [59,60].

The differences observed between years may reflect variation in seed lot or gastropod condition. Seeds used in both experiments were obtained from the same source but in different years and likely differed in maternal environment and physiological state, factors known to influence germination and early seedling responses [61–63]. For example, if seeds used in 2025 were older, we might expect less sensitivity to cues of herbivory risk, as predicted by error management theory [6]. Gastropods were also collected after experiencing different summer conditions prior to the experiments, and variation in temperature and moisture may have altered slug physiology and the chemical composition of their mucus [64,65]. Post-germination growing conditions also differed between years, with seedlings grown entirely in growth chambers in 2024 but transferred to greenhouse conditions in 2025, potentially affecting biomass responses [66]. Such sensitivity to environmental variation is expected in early life stages [67] and mirrors the conditions under which these interactions occur in nature.

Despite substantial interspecific and interannual variation, our results, together with previous research findings, suggest that gastropod mucus can provide chemical information associated with gastropod presence to *B. nigra.* However, the inconsistent responses across gastropod species and years suggest that there is not a uniform reaction to gastropod mucus. This is notable since these herbivore and plant species, including those examined in previous studies, vary in their native ranges and thus co-evolutionary history. Prior work with *C. aspersum* found that exposure to its mucus reduced seedling biomass and herbivore palatability in both *B. nigra* and *S. lycopersicum* [16,19], and gastropod mucus has also been shown to activate salicylic acid associated defense signaling in plants [20]. Increased germination speed in response to herbivore-associated cues has been documented in only two plant-herbivore systems: mucus from *Ar. subfuscus* on *B. nigra* [50,52] and *Ar. vulgaris* on *Capsicum annuum* [68]; the results of our 2024 experiment document similar responses to *Am. valentianus* and *C. aspersum* mucus.

Previous work has shown that gastropod mucus can activate PR1-associated defense signaling in plants [20]. However, identifying the mechanism underlying this response is complicated because locomotion mucus is not a single cue, but a complex mixture of compounds that may vary among gastropod species. For example, Kästner et al. detected salicylic acid in the mucus of *D. reticulatum* but not in 12 other gastropod species examined [69], illustrating that mucus composition can differ among species and may include compounds associated with plant signaling pathways [70]. Plant responses to any single compound can depend on the presence of other chemical, microbial, nutritional, or environmental stimuli because defense signaling is shaped by crosstalk among multiple hormonal pathways. Similar context dependence has been reported in snail-biocrust interactions, where mucus effects differ among snail species and may depend on interactions among mucus chemistry, consumer identity and microbial communities [71]. As a result, whole-mucus exposure cannot determine whether the responses observed here were caused by a specific kairomonal compound, general mucus chemistry, mucus-associated microbes, nutrients, salts, or interactions among these factors. Such chemical and contextual variation could contribute to the species-specific responses we observed. More broadly, the context-dependent nature of many plant-herbivore interactions [60] means that responses are often sensitive to variation in both biotic and abiotic factors. Herbivore pressure and associated plant demographic and trait responses frequently shift across years as a result of environmental variability [72,73]. Single-year experiments may therefore provide an incomplete picture of general patterns at both the species and functional group level [74], and temporally-replicated experiments have been identified as critically important in fields such as restoration ecology [75]. Yet such experiments remain relatively uncommon in studies of chemically mediated plant-herbivore interactions due to their logistical and methodological complexity. The differences observed between our two experimental years underscore the value of replication for evaluating the reliability of plant responses to herbivore-associated cues.

While our study provides new insights into plant-gastropod interactions, it is not without limitations. During the experiment, a high proportion (> 80%) of *B. nigra* seeds germinated; however, we did not continue observations after all treatments had reached a clear plateau. Although the proportion of treatment vs. control seeds germinating did not differ at this point, our data is therefore better suited to revealing differences in germination timing than final germination success. Because we focused on seeds and seedlings, we did not assess later fitness components like survival, reproduction, or competitive ability [76]. Herbivory can have longe-term consequences for plant performance, including transgenerational or maternal effects on offspring traits [77,78]. Future studies incorporating these metrics will be necessary to better understand the long-term consequences of exposure to gastropod mucus. Our experiments were also conducted under controlled conditions and therefore do not capture the full complexity of field environments where plants experience multiple, interacting biotic and abiotic factors. Field-based studies will be important for assessing the ecological relevance of our findings. Finally, we did not analyze the chemical composition of the mucus, which could provide critical insight into the mechanisms driving the observed plant responses. These responses could reflect specific kairomonal compounds, general mucus chemistry, mucus-associated microbes, nutrients, salts or other compounds present in mucus. Chemical profiling, combined with plant gene-expression assays, will be essential for determining which components of gastropod mucus function as cues or elicitors and for clarifying the mechanisms underlying these interactions.

Despite these limitations, our study contributes to a growing understanding of an often-overlooked component of plant-herbivore interactions. Our findings suggest that gastropod mucus cues can influence early plant traits and highlight the benefit of multi-year studies for better understanding the consistency of these responses. Continued research that integrates chemical analysis, broader taxonomic sampling, and field-based experiments will be essential for understanding how plants interpret and react to gastropod-derived cues, with potential implications for both natural and agricultural systems.
