# Supplementary material for: Herbivore-specific plant defense responses to a pre-attack risk cue
Source: PLoS One. 2026 Oct 7;21(10):e0358257. doi: 10.1371/journal.pone.0358257 (PMC13645048; doi:10.1371/journal.pone.0358257)
Supplement: Supplementary file 1 [file pone.0358257.s002.pdf]

August 11, 2026

Dear Dr. Albrectsen,

We thank you and the reviewer for your thoughtful and constructive comments on our manuscript. We appreciate the careful reading of our work and helpful suggestions for improving its clarity and interpretation. In revising the manuscript, we have focused on clarifying the distinction between germination timing and final germination success, moderating our interpretation of mucus as a gastropod-associated cue, improving the presentation of germination data, and adding methodological details when requested. Below we respond to each comment.

RESPONSE TO EDITOR

COMMENT 1

The experimental replication appears adequate for detecting moderate effects on germination timing. However, inspection of the cumulative germination curves presented in the Supplementary Material suggests that final germination was nearly complete across treatments, with differences occurring primarily in the timing of germination rather than in the final proportion of seeds germinating. I therefore encourage the authors to distinguish more clearly between effects on germination rate and effects on germination success. Some statements in the manuscript appear to imply effects on recruitment or germination success, whereas the data seem to support a more limited conclusion that certain mucus treatments, particularly Arion subfuscus, may accelerate germination under the experimental conditions used.

RESPONSE: We agree with this point and have revised the manuscript to be clear about the distinction between germination timing and germination success (l. 23 and 26-27 in the abstract, l. 244-48 in the results and l. 300-01 in the discussion). Each change clarifies that we interpret the germination results as effects on germination timing rather than germination percentage.

COMMENT 2

In addition, related to this point, could the authors justify whether the 48–50 h observation period was sufficient to capture final germination percentages rather than primarily differences in germination timing? Brassica nigra is a rapidly germinating species, but germination can still occur beyond this time window. If the observation period was insufficient to reach a clear plateau in all treatments, this limitation should be acknowledged.

RESPONSE: We now clarify this point in the methods: because the observation period was designed to capture the main phase of germination rather than to confirm absolute final germination after an extended plateau, our experiment provides a more effective test of germination timing rather than final germination success" (l. 170-73 in the methods and l. 367-71in the discussion).

COMMENT 3

The manuscript would benefit from clarification of the experimental unit used in the statistical analyses. Since seeds within a Petri dish share the same treatment environment, petri dish should be considered the independent experimental unit. Please clarify how this was handled particularly in the figure S1. For the seedling-growth experiment, only two seedlings per dish were assessed. This component of the study may have lower power to detect subtle treatment effects compared with the germination experiment. It would be useful for the authors to discuss this limitation when interpreting non-significant differences.

RESPONSE: We agree and now state more explicitly in the statistical analysis that we modeled each replicate plate as a random effect, (l. 213-14). Germination was therefore analyzed at the dish level, using the number of germinated seeds at each observation time, with repeated observations of each dish accounted for in the model. We also revised the germination figure caption (l. 267-68) to clarify that plotted values represent dish-level means across replicate Petri dishes rather than individual seeds. In the case of the seedling growth experiment, we grew two seeds from each of the replicate petri plates and analyzed the data using a linear mixed model that specified treatment as a fixed effect and box as a random effect.

COMMENT 4

The manuscript proposes that Brassica nigra may use mucus as an indicator of gastropod presence. This is an interesting hypothesis, but perhaps the authors could moderate the strength of this interpretation. The data demonstrate that exposure to mucus can alter germination dynamics. However, the experiment does not directly identify a causal compound involved, nor does it distinguish among alternative mechanisms. Consequently, the presence of kairomonal signaling (indicated in the introduction) should be presented as a hypothesis consistent with the results. Similarly, I found the statement that "mucus may serve as a generalized indicator of gastropod presence for B. nigra" difficult to interpret. Given the interspecific and interannual variation, it would be helpful if the authors clarified what is meant by "generalized indicator" and more clearly distinguished the observed response from the broader ecological interpretation.

RESPONSE: We have revised the manuscript to make our interpretation of mucus as a gastropod-associated cue more cautious (l. 72-73 in the introduction) and state in the discussion that our data suggests that gastropod mucus can provide chemical information associated with gastropod presence for B. nigra. However, the inconsistent responses across gastropod species and years, suggest that there is not a uniform reaction to gastropod mucus (l.328-31).

COMMENT 5

The cumulative germination curves presented in the Supplementary Material are highly informative and, in my opinion, more biologically meaningful than the figures showing differences relative to the control treatment. I strongly encourage the authors to move the cumulative germination curves into the main manuscript. Presenting cumulative germination trajectories for each year as the primary figure and retain the control-subtracted (treatment – control) presentation as an accompanying figure, would allow readers to assess both the overall germination dynamics and the magnitude of treatment effects. You may also wish to consider whether continuous lines are the most appropriate visualisation of the subtracted values, or whether alternative representations of differences in germination status through time could improve readability.

RESPONSE: We moved the cumulative germination curves from the supplemental material into the main manuscript as the new Figure 1. The original Figure 1 is now Fig 2 and original Fig 2 is now Fig 3.

REVIEWER COMMENTS

We thank the reviewer for the thoughtful and constructive comments. We appreciate the reviewer's careful attention to the conceptual framing of the manuscript, the mechanistic interpretation of our results, and the clarity of the statistical methods. Several of these comments overlapped with those from the editor and we made changes that addressed them simultaneously in revising the manuscript. Below we respond to each specific comment.

COMMENT 1

The central conclusion of the manuscript is that plant responses to gastropod mucus are variable and context-dependent. While this interpretation is supported by the data, the manuscript alternates between presenting mucus as a generalized herbivore cue and emphasizing the inconsistency and species-specificity of responses. These two perspectives are not fully reconciled. The authors should clarify whether gastropod mucus is best interpreted as a broadly reliable but environmentally modulated signal, or as a cue that plants respond to selectively depending on herbivore identity and context. Providing a clearer conceptual framing would strengthen the overall interpretation.

RESPONSE: We have revised the manuscript to clarify our conceptual framing. Our intention was not to suggest that gastropod mucus functions as a uniformly reliable signal across all gastropod species or environmental contexts. Rather, we interpret mucus as a potential herbivore-associated cue to which plants may respond selectively depending on herbivore identity and context. As mentioned in our response to the editor, we have therefore revised both the introduction (l. 72-73) and the discussion (l.328-31) to note that inconsistent changes in germination timing suggest that it is not a uniform reaction. The changes better reconcile the broader ecological interpretation of mucus as a potential herbivore-associated cue with the species specific and year-dependent responses observed in our study.

COMMENT 2

The manuscript is strong in documenting ecological patterns, but mechanistic insight remains limited. While the Discussion notes that mucus chemistry likely varies among species, this point is not explored in depth. The authors should expand their discussion of potential mechanisms, including known components of gastropod mucus (e.g., salicylic acid in some species) and how these might relate to plant defense signaling pathways. A clearer connection between observed phenotypic responses and underlying molecular mechanisms would enhance the impact of the study.

RESPONSE: We have revised the manuscript to provide a clearer connection between the observed phenotypic responses and possible underlying mechanisms (l.341-55). Specifically, we expanded the connection between our results and previous work showing that gastropod mucus can activate PR-associated defense signaling in plants. We also discuss evidence that mucus chemistry varies among gastropod species, including prior work showing that salicylic acid was detected in the mucus of *D. reticulatum* but not in several other gastropod species. We added that chemical and contextual variation could contribute to the species-specific responses observed in our experiment and included additional support for this interpretation. Finally, we clarified that because mucus is a complex mixture, our experiment did not identify the active compounds or directly test the signaling pathways involved, and that

identifying the specific compounds responsible for these responses will require chemical profiling of gastropod mucus, ideally combined with plant gene-expression assays (l. 380-83).

COMMENT 3

The statistical analyses are generally appropriate and well justified, particularly the shift from Cox proportional hazards models to generalized linear mixed models due to violations of assumptions. However, some clarification would improve transparency. The authors should briefly describe how overdispersion was assessed in the binomial models, clarify whether any corrections for multiple comparisons were applied beyond Dunnett's test, and provide a short rationale for not pursuing alternative survival analysis approaches after assumption violations.

RESPONSE: We have added text to the revised ms. to describe how we assessed dispersion (using simulations provided in the DHARMa package) (l. 217-20), and we have added text to provide dispersion statistics and simulation-based p-values for each of our final GLMM models) (l.247-48 and 256-57. We have also added text to clarify that we u. Tukey tests when conducting pairwise linear contrasts following significant main effects in our GLMM models (l. 218-20). We have also added text to the revised ms. to provide details about the additional alternative survival analysis approaches we explored once the proportional hazards assumption was violated, including accelerated failure-time models and restricted mean survival time analyses (l.198-206).

Sincerely,

Eirette Santiago

on behalf of the authors
