# Supplementary material for: Herbivore-specific plant defense responses to a pre-attack risk cue
Source: PLoS One. 2026 Oct 7;21(10):e0358257. doi: 10.1371/journal.pone.0358257 (PMC13645048; doi:10.1371/journal.pone.0358257)
Supplement: Peer review history

# Peer review: Decision Letter 0

**DOI:** 10.1371/journal.pone.0358257.r001

**Authors:** Benedicte Riber Albrectsen

16 Jun 2026

Dear Dr. Santiago,

Thank you for submitting your manuscript to PLOS ONE. After careful consideration, we feel that it has merit but does not fully meet PLOS ONE’s publication criteria as it currently stands. Therefore, we invite you to submit a revised version of the manuscript that addresses the points raised during the review process.

Recommendations are provided below from one reviewer and myself. Both find that the study is carefully conducted and clearly presented, and inclusion of the second experimental year is appreciated. Addressing the points would further strengthen the manuscript and help ensure that the conclusions remain closely aligned with the evidence presented.

Please submit your revised manuscript by Jul 31 2026 11:59PM. If you will need more time than this to complete your revisions, please reply to this message or contact the journal office at plosone@plos.org. When you're ready to submit your revision, log on to https://www.editorialmanager.com/pone/ and select the 'Submissions Needing Revision' folder to locate your manuscript file.

- A letter that responds to each point raised by the academic editor and reviewer(s). You should upload this letter as a separate file labeled 'Response to Reviewers'.
- A marked-up copy of your manuscript that highlights changes made to the original version. You should upload this as a separate file labeled 'Revised Manuscript with Track Changes'.
- An unmarked version of your revised paper without tracked changes. You should upload this as a separate file labeled 'Manuscript'.

If you would like to make changes to your financial disclosure, please include your updated statement in your cover letter. Guidelines for resubmitting your figure files are available below the reviewer comments at the end of this letter.

If applicable, we recommend that you deposit your laboratory protocols in protocols.io to enhance the reproducibility of your results. Protocols.io assigns your protocol its own identifier (DOI) so that it can be cited independently in the future. For instructions see: https://journals.plos.org/plosone/s/submission-guidelines#loc-laboratory-protocols. Additionally, PLOS ONE offers an option for publishing peer-reviewed Lab Protocol articles, which describe protocols hosted on protocols.io. Read more information on sharing protocols at https://plos.org/protocols?utm_medium=editorial-email&utm_source=authorletters&utm_campaign=protocols.

As the corresponding author, your ORCID iD is verified in the submission system and will appear in the published article. PLOS supports the use of ORCID, and we encourage all coauthors to register for an ORCID iD and use it as well. Please encourage your coauthors to verify their ORCID iD within the submission system before final acceptance, as unverified ORCID iDs will not appear in the published article. *Only*  the individual author can complete the verification step; PLOS staff *cannot*  verify ORCID iDs on behalf of authors.

We look forward to receiving your revised manuscript.

Kind regards,

Benedicte Riber Albrectsen

Academic Editor

PLOS One

Journal Requirements:

When submitting your revision, we need you to address these additional requirements.

1. Please ensure that your manuscript meets PLOS ONE's style requirements, including those for file naming. The PLOS ONE style templates can be found at

https://journals.plos.org/plosone/s/file?id=wjVg/PLOSOne_formatting_sample_main_body.pdf and

https://journals.plos.org/plosone/s/file?id=ba62/PLOSOne_formatting_sample_title_authors_affiliations.pdf.

2.In your Methods section, please provide additional information regarding the permits you obtained for the work. Please ensure you have included the full name of the authority that approved the field site access and, if no permits were required, a brief statement explaining why

3. If the reviewer comments include a recommendation to cite specific previously published works, please review and evaluate these publications to determine whether they are relevant and should be cited. There is no requirement to cite these works unless the editor has indicated otherwise.

4. Please review your reference list to ensure that it is complete and correct. If you have cited papers that have been retracted, please include the rationale for doing so in the manuscript text, or remove these references and replace them with relevant current references. Any changes to the reference list should be mentioned in the rebuttal letter that accompanies your revised manuscript. If you need to cite a retracted article, indicate the article’s retracted status in the References list and also include a citation and full reference for the retraction notice.

Additional Editor Comments (if provided):

1. The experimental replication appears adequate for detecting moderate effects on germination timing. However, inspection of the cumulative germination curves presented in the Supplementary Material suggests that final germination was nearly complete across treatments, with differences occurring primarily in the timing of germination rather than in the final proportion of seeds germinating. I therefore encourage the authors to distinguish more clearly between effects on germination rate and effects on germination success. Some statements in the manuscript appear to imply effects on recruitment or germination success, whereas the data seem to support a more limited conclusion that certain mucus treatments, particularly Arion subfuscus, may accelerate germination under the experimental conditions used.

In addition, related to this point, could the authors justify whether the 48–50 h observation period was sufficient to capture final germination percentages rather than primarily differences in germination timing? Brassica nigra is a rapidly germinating species, but germination can still occur beyond this time window. If the observation period was insufficient to reach a clear plateau in all treatments, this limitation should be acknowledged.

2. Experimental unit and statistical analysis

The manuscript would benefit from clarification of the experimental unit used in the statistical analyses. Since seeds within a Petri dish share the same treatment environment, petri dish should be considered the independent experimental unit. Please clarify how this was handled particularily in the figure S1. For the seedling-growth experiment, only two seedlings per dish were assessed. This component of the study may have lower power to detect subtle treatment effects compared with the germination experiment. It would be useful for the authors to discuss this limitation when interpreting non-significant differences.

3. Interpretation of mucus as a gastropod cue

The manuscript proposes that Brassica nigra may use mucus as an indicator of gastropod presence. This is an interesting hypothesis, but perhaps the authors could moderate the strength of this interpretation. The data demonstrate that exposure to mucus can alter germination dynamics. However, the experiment does not directly identify a causal compounds involved, nor does it distinguish among alternative mechanisms. Consequently, the presence of kairomonal signalling (indicated in the introduction) should be presented as a hypothesis consistent with the results. Similarly, I found the statement that “mucus may serve as a generalized indicator of gastropod presence for B. nigra” difficult to interpret. Given the interspecific and interannual variation, it would be helpful if the authors clarified what is meant by “generalized indicator” and more clearly distinguished the observed response from the broader ecological interpretation.

4. Presentation of germination data

The cumulative germination curves presented in the Supplementary Material are highly informative and, in my opinion, more biologically meaningful than the figures showing differences relative to the control treatment. I strongly encourage the authors to move the cumulative germination curves into the main manuscript.

Presenting cumulative germination trajectories for each year as the primary figure and retain the control-subtracted (treatment – control) presentation as an accompanying figure, would allow readers to assess both the overall germination dynamics and the magnitude of treatment effects.

You may also wish to consider whether continuous lines are the most appropriate visualisation of the subtracted values, or whether alternative representations of differences in germination status through time could improve readability.

[Note: HTML markup is below. Please do not edit.]

Reviewers' comments:

Reviewer's Responses to Questions

**Comments to the Author**

1. Is the manuscript technically sound, and do the data support the conclusions?

Reviewer #1: Yes

**********

2. Has the statistical analysis been performed appropriately and rigorously?

Reviewer #1: Yes

**********

3. Have the authors made all data underlying the findings in their manuscript fully available?

The PLOS Data policy

Reviewer #1: Yes

**********

4. Is the manuscript presented in an intelligible fashion and written in standard English?

Reviewer #1: Yes

**********

Reviewer #1: Review report

This manuscript examines whether Brassica nigra can detect and respond to herbivore-associated pre-attack cues, specifically gastropod locomotion mucus, across multiple herbivore species and two experimental years. The study addresses an important and timely question in plant–herbivore ecology concerning the ability of plants to anticipate herbivory risk and adjust early life-history traits accordingly. The experimental design is robust, particularly due to the inclusion of multiple gastropod species and temporal replication, which enhances ecological relevance and addresses a common limitation in this field. The results are clearly presented and demonstrate that plant responses are highly context-dependent, varying across herbivore species and between years. Overall, the manuscript is scientifically sound and suitable for publication following minor revisions focused on clarity, interpretation, and methodological detail.

1. The central conclusion of the manuscript is that plant responses to gastropod mucus are variable and context-dependent. While this interpretation is supported by the data, the manuscript alternates between presenting mucus as a generalized herbivore cue and emphasizing the inconsistency and species-specificity of responses. These two perspectives are not fully reconciled. The authors should clarify whether gastropod mucus is best interpreted as a broadly reliable but environmentally modulated signal, or as a cue that plants respond to selectively depending on herbivore identity and context. Providing a clearer conceptual framing would strengthen the overall interpretation.

2. The manuscript is strong in documenting ecological patterns, but mechanistic insight remains limited. While the Discussion notes that mucus chemistry likely varies among species, this point is not explored in depth. The authors should expand their discussion of potential mechanisms, including known components of gastropod mucus (e.g., salicylic acid in some species) and how these might relate to plant defense signaling pathways. A clearer connection between observed phenotypic responses and underlying molecular mechanisms would enhance the impact of the study.

3. The statistical analyses are generally appropriate and well justified, particularly the shift from Cox proportional hazards models to generalized linear mixed models due to violations of assumptions. However, some clarification would improve transparency. The authors should briefly describe how overdispersion was assessed in the binomial models, clarify whether any corrections for multiple comparisons were applied beyond Dunnett’s test, and provide a short rationale for not pursuing alternative survival analysis approaches after assumption violations.

**********

what does this mean?). If published, this will include your full peer review and any attached files.

If you choose “no”, your identity will remain anonymous but your review may still be made public.

**Do you want your identity to be public for this peer review?**  For information about this choice, including consent withdrawal, please see our Privacy Policy

Reviewer #1: No

**********

[NOTE: If reviewer comments were submitted as an attachment file, they will be attached to this email and accessible via the submission site. Please log into your account, locate the manuscript record, and check for the action link "View Attachments". If this link does not appear, there are no attachment files.]

To ensure your figures meet our technical requirements, please review our figure guidelines: https://journals.plos.org/plosone/s/figures

You may also use PLOS’s free figure tool, NAAS, to help you prepare publication quality figures: https://journals.plos.org/plosone/s/figures#loc-tools-for-figure-preparation.

NAAS will assess whether your figures meet our technical requirements by comparing each figure against our figure specifications.

# Author comment: Author response to Decision Letter 1

**DOI:** 10.1371/journal.pone.0358257.r002

12 Aug 2026

August 11, 2026

Dear Dr. Albrectsen,

We thank you and the reviewer for your thoughtful and constructive comments on our manuscript. We appreciate the careful reading of our work and helpful suggestions for improving its clarity and interpretation. In revising the manuscript, we have focused on clarifying the distinction between germination timing and final germination success, moderating our interpretation of mucus as a gastropod-associated cue, improving the presentation of germination data, and adding methodological details when requested. Below we respond to each comment.

RESPONSE TO EDITOR

COMMENT 1

The experimental replication appears adequate for detecting moderate effects on germination timing. However, inspection of the cumulative germination curves presented in the Supplementary Material suggests that final germination was nearly complete across treatments, with differences occurring primarily in the timing of germination rather than in the final proportion of seeds germinating. I therefore encourage the authors to distinguish more clearly between effects on germination rate and effects on germination success. Some statements in the manuscript appear to imply effects on recruitment or germination success, whereas the data seem to support a more limited conclusion that certain mucus treatments, particularly Arion subfuscus, may accelerate germination under the experimental conditions used.

RESPONSE: We agree with this point and have revised the manuscript to be clear about the distinction between germination timing and germination success (l. 23 and 26-27 in the abstract, l. 244-48 in the results and l. 300-01 in the discussion). Each change clarifies that we interpret the germination results as effects on germination timing rather than germination percentage.

COMMENT 2

In addition, related to this point, could the authors justify whether the 48–50 h observation period was sufficient to capture final germination percentages rather than primarily differences in germination timing? Brassica nigra is a rapidly germinating species, but germination can still occur beyond this time window. If the observation period was insufficient to reach a clear plateau in all treatments, this limitation should be acknowledged.

RESPONSE: We now clarify this point in the methods: because the observation period was designed to capture the main phase of germination rather than to confirm absolute final germination after an extended plateau, our experiment provides a more effective test of germination timing rather than final germination success” (l. 170-73 in the methods and l. 367-71in the discussion).

COMMENT 3

The manuscript would benefit from clarification of the experimental unit used in the statistical analyses. Since seeds within a Petri dish share the same treatment environment, petri dish should be considered the independent experimental unit. Please clarify how this was handled particularly in the figure S1. For the seedling-growth experiment, only two seedlings per dish were assessed. This component of the study may have lower power to detect subtle treatment effects compared with the germination experiment. It would be useful for the authors to discuss this limitation when interpreting non-significant differences.

RESPONSE: We agree and now state more explicitly in the statistical analysis that we modeled each replicate plate as a random effect, (l. 213-14). Germination was therefore analyzed at the dish level, using the number of germinated seeds at each observation time, with repeated observations of each dish accounted for in the model. We also revised the germination figure caption (l. 267-68) to clarify that plotted values represent dish-level means across replicate Petri dishes rather than individual seeds. In the case of the seedling growth experiment, we grew two seeds from each of the replicate petri plates and analyzed the data using a linear mixed model that specified treatment as a fixed effect and box as a random effect.

COMMENT 4

The manuscript proposes that Brassica nigra may use mucus as an indicator of gastropod presence. This is an interesting hypothesis, but perhaps the authors could moderate the strength of this interpretation. The data demonstrate that exposure to mucus can alter germination dynamics. However, the experiment does not directly identify a causal compound involved, nor does it distinguish among alternative mechanisms. Consequently, the presence of kairomonal signaling (indicated in the introduction) should be presented as a hypothesis consistent with the results. Similarly, I found the statement that “mucus may serve as a generalized indicator of gastropod presence for B. nigra” difficult to interpret. Given the interspecific and interannual variation, it would be helpful if the authors clarified what is meant by “generalized indicator” and more clearly distinguished the observed response from the broader ecological interpretation.

RESPONSE: We have revised the manuscript to make our interpretation of mucus as a gastropod-associated cue more cautious (l. 72-73 in the introduction) and state in the discussion that our data suggests that gastropod mucus can provide chemical information associated with gastropod presence for B. nigra. However, the inconsistent responses across gastropod species and years, suggest that there is not a uniform reaction to gastropod mucus (l.328-31).

COMMENT 5

The cumulative germination curves presented in the Supplementary Material are highly informative and, in my opinion, more biologically meaningful than the figures showing differences relative to the control treatment. I strongly encourage the authors to move the cumulative germination curves into the main manuscript. Presenting cumulative germination trajectories for each year as the primary figure and retain the control-subtracted (treatment – control) presentation as an accompanying figure, would allow readers to assess both the overall germination dynamics and the magnitude of treatment effects. You may also wish to consider whether continuous lines are the most appropriate visualisation of the subtracted values, or whether alternative representations of differences in germination status through time could improve readability.

RESPONSE: We moved the cumulative germination curves from the supplemental material into the main manuscript as the new Figure 1. The original Figure 1 is now Fig 2 and original Fig 2 is now Fig 3.

REVIEWER COMMENTS

We thank the reviewer for the thoughtful and constructive comments. We appreciate the reviewer’s careful attention to the conceptual framing of the manuscript, the mechanistic interpretation of our results, and the clarity of the statistical methods. Several of these comments overlapped with those from the editor and we made changes that addressed them simultaneously in revising the manuscript. Below we respond to each specific comment.

COMMENT 1

The central conclusion of the manuscript is that plant responses to gastropod mucus are variable and context-dependent. While this interpretation is supported by the data, the manuscript alternates between presenting mucus as a generalized herbivore cue and emphasizing the inconsistency and species-specificity of responses. These two perspectives are not fully reconciled. The authors should clarify whether gastropod mucus is best interpreted as a broadly reliable but environmentally modulated signal, or as a cue that plants respond to selectively depending on herbivore identity and context. Providing a clearer conceptual framing would strengthen the overall interpretation.

RESPONSE: We have revised the manuscript to clarify our conceptual framing. Our intention was not to suggest that gastropod mucus functions as a uniformly reliable signal across all gastropod species or environmental contexts. Rather, we interpret mucus as a potential herbivore-associated cue to which plants may respond selectively depending on herbivore identity and context. As mentioned in our response to the editor, we have therefore revised both the introduction (l. 72-73) and the discussion (l.328-31) to note that inconsistent changes in germination timing suggest that it is not a uniform reaction. The changes better reconcile the broader ecological interpretation of mucus as a potential herbivore-associated cue with the species specific and year-dependent responses observed in our study.

COMMENT 2

The manuscript is strong in documenting ecological patterns, but mechanistic insight remains limited. While the Discussion notes that mucus chemistry likely varies among species, this point is not explored in depth. The authors should expand their discussion of potential mechanisms, including known components of gastropod mucus (e.g., salicylic acid in some species) and how these might relate to plant defense signaling pathways. A clearer connection between observed phenotypic responses and underlying molecular mechanisms would enhance the impact of the study.

RESPONSE: We have revised the manuscript to provide a clearer connection between the observed phenotypic responses and possible underlying mechanisms (l.341-55). Specifically, we expanded the connection between our results and previous work showing that gastropod mucus can activate PR-associated defense signaling in plants. We also discuss evidence that mucus chemistry varies among gastropod species, including prior work showing that salicylic acid was detected in the mucus of D. reticulatum but not in several other gastropod species. We added that chemical and contextual variation could contribute to the species-specific responses observed in our experiment and included additional support for this interpretation. Finally, we clarified that because mucus is a complex mixture, our experiment did not identify the active compounds or directly test the signaling pathways involved, and that identifying the specific compounds responsible for these responses will require chemical profiling of gastropod mucus, ideally combined with plant gene-expression assays (l. 380-83).

COMMENT 3

The statistical analyses are generally appropriate and well justified, particularly the shift from Cox proportional hazards models to generalized linear mixed models due to violations of assumptions. However, some clarification would improve transparency. The authors should briefly describe how overdispersion was assessed in the binomial models, clarify whether any corrections for multiple comparisons were applied beyond Dunnett’s test, and provide a short rationale for not pursuing alternative survival analysis approaches after assumption violations.

RESPONSE: We have added text to the revised ms. to describe how we assessed dispersion (using simulations provided in the DHARMa package) (l. 217-20), and we have added text to provide dispersion statistics and simulation-based p-values for each of our final GLMM models) (l.247-48 and 256-57. We have also added text to clarify that we u. Tukey tests when conducting pairwise linear contrasts following significant main effects in our GLMM models (l. 218-20). We have also added text to the revised ms. to provide details about the additional alternative survival analysis approaches we explored once the proportional hazards assumption was violated, including accelerated failure-time models and restricted mean survival time analyses (l.198-206).

Sincerely,

Eirette Santiago

on behalf of the authors

Attachment Submitted filename: Response to Reviewers.pdf

# Editor report: Decision Letter 1

**DOI:** 10.1371/journal.pone.0358257.r003

**Authors:** Benedicte Riber Albrectsen

31 Aug 2026

Herbivore-specific plant defense responses to a pre-attack risk cue

PONE-D-26-13822R1

Dear Dr. Santiago,

We’re pleased to inform you that your manuscript has been judged scientifically suitable for publication and will be formally accepted for publication once it meets all outstanding technical requirements.

Within one week, you’ll receive an e-mail detailing the required amendments. When these have been addressed, you’ll receive a formal acceptance letter and your manuscript will be scheduled for publication.

An invoice will be generated when your article is formally accepted. Please note, if your institution has a publishing partnership with PLOS and your article meets the relevant criteria, all or part of your publication costs will be covered. Please make sure your user information is up-to-date by logging into Editorial Manager at Editorial Manager® and clicking the ‘Update My Information' link at the top of the page. For questions related to billing, please contact billing support.

If your institution or institutions have a press office, please notify them about your upcoming paper to help maximize its impact. If they’ll be preparing press materials, please inform our press team as soon as possible -- no later than 48 hours after receiving the formal acceptance. Your manuscript will remain under strict press embargo until 2 pm Eastern Time on the date of publication. For more information, please contact onepress@plos.org.

Kind regards,

Benedicte Riber Albrectsen

Academic Editor

PLOS One

Additional Editor Comments (optional):

The reviewers' comments and concerns have been satisfactorily addressed in a detailed point-by-point response, and the manuscript has been revised accordingly.

Reviewers' comments:

# Editor report: Acceptance letter

**DOI:** 10.1371/journal.pone.0358257.r004

**Authors:** Benedicte Riber Albrectsen

PONE-D-26-13822R1

PLOS One

Dear Dr. Santiago,

I'm pleased to inform you that your manuscript has been deemed suitable for publication in PLOS One. Congratulations! Your manuscript is now being handed over to our production team.

At this stage, our production department will prepare your paper for publication. This includes ensuring the following:

* All references, tables, and figures are properly cited

* All relevant supporting information is included in the manuscript submission,

* There are no issues that prevent the paper from being properly typeset

You will receive further instructions from the production team, including instructions on how to review your proof when it is ready. Please keep in mind that we are working through a large volume of accepted articles, so please give us a few days to review your paper and let you know the next and final steps.

Lastly, if your institution or institutions have a press office, please let them know about your upcoming paper now to help maximize its impact. If they'll be preparing press materials, please inform our press team within the next 48 hours. Your manuscript will remain under strict press embargo until 2 pm Eastern Time on the date of publication. For more information, please contact onepress@plos.org.

You will receive an invoice from PLOS for your publication fee after your manuscript has reached the completed accept phase. If you receive an email requesting payment before acceptance or for any other service, this may be a phishing scheme. Learn how to identify phishing emails and protect your accounts at https://explore.plos.org/phishing.

If we can help with anything else, please email us at customercare@plos.org.

Thank you for submitting your work to PLOS One and supporting open access.

Kind regards,

PLOS One Editorial Office Staff

on behalf of

Dr. Benedicte Riber Albrectsen

Academic Editor

PLOS One
